# Rab21 recruits EEA1 and competes with Rab5 for Rabex-5 activation

**DOI:** 10.3389/fcell.2025.1588308

**Published:** 2025-05-30

**Authors:** Francisco Yanguas, Cinzia Progida

**Affiliations:** Department of Biosciences, University of Oslo, Oslo, Norway

**Keywords:** RABs GTPases, endosome, protein trafficking, GEFs (guanine nucleotide exchange factors), Rab5, Rab21, RABEX-5, EEA1

## Abstract

Rab5 is a key regulator of early endosomal traffic and fusion. It shares its localization and guanine nucleotide exchange factor Rabex-5 with the less characterized member of the Rab5 subfamily Rab21. Here, we found that, similarly to Rab5, Rab21 also interacts with the tethering protein EEA1. Overexpression of Rab21 rescues the defects in EEA1 localization and endosomal size caused by the depletion of PI3P or the inhibition of Rab5 function, both needed for the recruitment of EEA1 to early endosomes. Interestingly, modulation of the binding properties of Rab5 or Rab21 dominant negative mutant with Rabex-5 support a model in which Rab5 and Rab21 compete for the activation by Rabex-5 and suggest that Rab21 might have higher affinity for this GEF than Rab5 *in vivo*. Altogether, our results reveal that Rab21 regulates early endosomal size by recruiting EEA1 to the endosomes via a pathway parallel to Rab5 and highlight Rabex-5’s critical role in Rab21 and Rab5 cross-regulation.

## Introduction

Rab proteins constitute a family of small GTPases that regulate intracellular membrane traffic. They give identity to compartments and participate in different steps of vesicular transport by acting as molecular switches (Wandinger-Ness and Zerial, 2014). These proteins are active when they are bound to GTP and inactive when bound to GDP. Their activation is facilitated by Guanine nucleotide Exchange Factors (GEFs) that mediate the exchange of GDP by GTP, while the inactive state is induced by GTPase Activating Proteins (GAPs) that stimulate the intrinsic GTPase activity of these small GTPases (Lamber et al., 2019). When active, Rabs are normally membrane-bound and mediates different steps of protein trafficking by interacting with various effectors such as coats, tethers, enzymes, motors and cytoskeleton proteins (Hutagalung and Novick, 2011). Upon inactivation, they detach from the membrane (Wilmes and Kümmel, 2023).

Different cellular organelles possess distinct Rabs composition (Wandinger-Ness and Zerial, 2014). Early endosomes (EEs) are important compartments for protein sorting, delivering cargos either to the plasma membrane, to the biosynthetic pathway via the *trans* Golgi network (TGN) for recycling, or to lysosomes for degradation by maturing into late endosomes (Naslavsky and Caplan, 2018; Elkin et al., 2016). Rab5 and Rab21 are members of the Rab5 subfamily and localize to EEs where they regulate early endocytic pathways (Simpson et al., 2004; Gorvel et al., 1991; Stenmark et al., 1994; Bucci et al., 1992).

Rab5 is considered a master regulator of EE traffic regulating different processes such as endocytosis, endosomal fusion, and endosomal maturation (Gorvel et al., 1991; Stenmark et al., 1994; Bucci et al., 1992; Rink et al., 2005; Poteryaev et al., 2010). Rab5 regulates endosomal fusion and size by recruiting and directly interacting with the tethering factors Rabenosyn-5 and Early Endosome Antigen 1 (EEA1) and by regulating phosphatidylinositol 3-phosphate (PI3P) synthesis (Nielsen et al., 2000; Christoforidis et al., 1999; Murray et al., 2002; Tremel et al., 2021; Callaghan et al., 1999a; Simonsen et al., 1998; Lawe et al., 2002; McBride et al., 1999, 13). EEA1 is a long coiled-coil protein that acts as an homodimer (Callaghan et al., 1999b). It is composed by an N-terminal zinc finger domain that binds directly to Rab5, followed by a coiled-coil region with a homodimerization site and another Rab5 binding site close to the Fab-1, YGL023, Vps27, and EEA1 (FYVE) domain at the C-terminal of the protein (Simonsen et al., 1998; Callaghan et al., 1999b; Dumas et al., 2001). The FYVE domain, by interacting with PI3P, is responsible for the recruitment of EEA1 to the EE membrane (Gaullier et al., 1998; Patki et al., 1998; Patki et al., 1997).

Rab21 is implicated in retromer-mediated recycling to the plasma membrane by facilitating tubulation at EEs (Del Olmo et al., 2019b; Pei et al., 2023). Additionally, Rab21 participates in endocytosis regulating caveolin-mediated internalization and integrin endocytosis in a clathrin-independent pathway (Moreno-Layseca et al., 2021; Shikanai et al., 2023). Rab21 is also present at the Golgi complex where it sorts Vamp7 to the cell periphery and facilitates TMED10 localization at the Golgi complex (Simpson et al., 2004; Del Olmo et al., 2019a; Burgo et al., 2012; Constantino-Jonapa et al., 2020).

Rab5 and Rab21 can be activated by different GEFs. For example, Als2 and Rin1 activate Rab5 while Varp is a GEF for Rab21 (Zhang et al., 2006; Tall et al., 2001; Topp et al., 2004). In addition, Rabex-5 is a EE protein that acts as GEF for both Rab5 and Rab21 (Delprato et al., 2004; Delprato and Lambright, 2007; Horiuchi et al., 1997; Lippé et al., 2001). Its function is important to stimulate EE fusion mediated by Rab5 and for the trafficking of ubiquitinated proteins to the lysosome (Horiuchi et al., 1997; Lippé et al., 2001; Penengo et al., 2006; Mattera and Bonifacino, 2008; Aikawa, 2012; Aikawa et al., 2012).

While the function of Rab5 at EE has been broadly studied and its role in regulating EE fusion is well established, the function of Rab21 is less characterized, although it has a role in the endocytosis of transferrin, integrins and epidermal growth factor (EGF) (Simpson et al., 2004; Moreno-Layseca et al., 2021; Yang et al., 2012) and is involved in different human diseases (Li et al., 2023). The data available show that, similarly to Rab5, the expression of a dominant negative (DN) mutant of Rab21, which is locked in the inactive form, reduces EEs size while the overexpression of the wild-type (WT) version induces EE enlargement (Simpson et al., 2004; Dinneen and Ceresa, 2004). However, how Rab21 regulates this process is not fully understood. Thus, in this work we investigated how Rab21 regulates EE size and its relationship with Rab5. Our results show that Rab21 is able to recruit EEA1 on membranes independently of Rab5 function. In addition, our data indicate that the expression of Rab21 DN mislocalizes Rab5 to the Golgi apparatus and that this is due to a competition between Rab5 and Rab21 for the activation by the GEF Rabex-5. Altogether, our results reveal that Rab21 regulates EE size by recruiting EEA1 to the endosomes through a route parallel to Rab5’s route and point out that Rabex-5 is a critical player in the cross-regulation between Rab21 and Rab5.

## Results

### The dominant negative mutant of Rab21 reduces membrane recruitment of EEA1 and affects the formation of early endosomes

**FIGURE 1:**
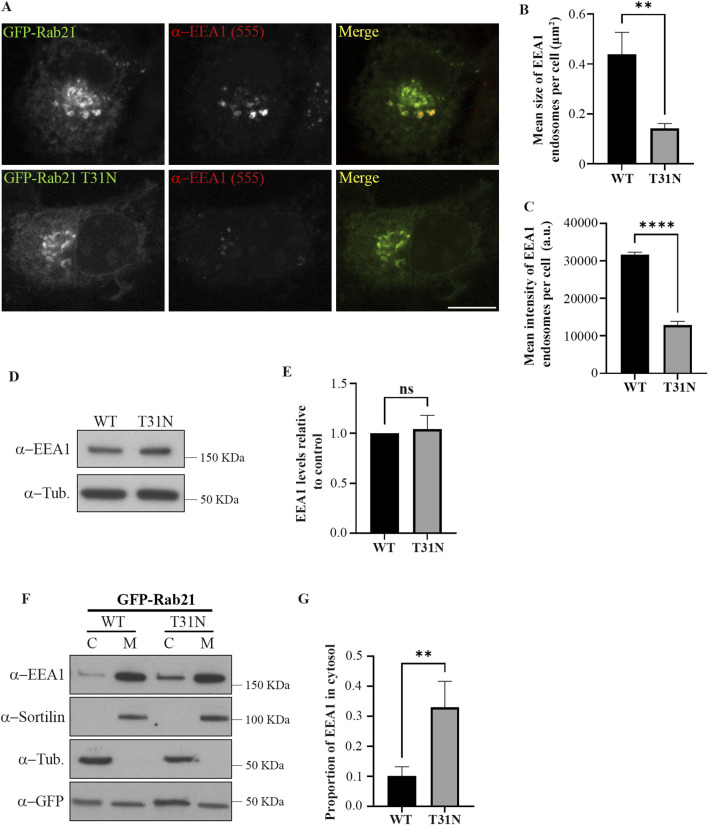
The dominant negative mutant of Rab21 reduces EE size and EEA1 recruitment to the membranes. **(A)** Representative images of N2a cells transfected with GFP-Rab21 WT or GFP-Rab21 T31N, and immunostained with an antibody against EEA1. Scale bar: 10 µm. Quantification of the mean size of EEA1-positive endosomes per cell **(B)** and of the mean intensity of EEA1-positive endosome per cell **(C)** in cells transfected with GFP-Rab21 WT or GFP-Rab21 T31N. The graphs represent the mean and standard deviation of three independent experiments. n ≥ 58 cells per condition. **(D)** Cell lysates from N2a cells transfected with GFP-Rab21 WT or T31N were subjected to Western blot analysis with antibodies against EEA1 and tubulin as loading control. **(E)** Quantification of EEA1 protein levels relative to the level of tubulin and normalized to the sample transfected with GFP-Rab21 WT. The graph represents the mean and standard deviation of three independent experiments. **(F)** Cell lysates from N2a cells transfected with GFP-Rab21 WT or T31N were subjected to subcellular fractionation and the membrane and cytosolic fractions were subjected to Western blot analysis with antibodies against EEA1, sortilin, tubulin and GFP. sortilin and tubulin were used as a control to confirm efficient membrane and cytoplasm fractions separation, respectively. **(G)** Quantification of the proportion of EEA1 present in the cytosol relative to the total. The graph shows the mean and standard deviation of four independent experiments. For statistical analysis in b,c, e and g, a Student’s t-test was performed. **P < 0.01; ****P < 0,0001. ns: not significant; C: cytosol; M: membrane; a.u.: arbitrary units.

To study the role of Rab21 at early endosomes (EEs), we first analyzed EE distribution by immunostaining EEA1 in Neuro2a cells expressing GFP-tagged Rab21 WT or dominant negative (DN) mutant T31N. As shown in Figure 1A, when expressing GFP-Rab21 T31N, EEA1 endosomes have a 3-fold reduced mean size per cell compared to cells expressing GFP-Rab21 WT and a 2,5-fold decrease in mean intensity (Figures 1A–C). This alteration is not due to a reduction in EEA1 expression, as shown in Figures 1D,E, and it is in agreement with previous observations in HeLa cells (Simpson et al., 2004; Supplementary Figure S1).

Next, we wondered if the alteration of EEA1-positive endosomes caused by the expression of Rab21 DN mutant could be due to a defect in EEA1 recruitment to EEs. To study this possibility, we separated the membranes from the cytosolic fraction in cells transfected with GFP-Rab21 WT or GFP-Rab21 T31N and determined the distribution of EEA1 between these fractions (Figures 1F,G). In line with the microscopy experiments, the results indicate that the expression of Rab21 T31N reduces EEA1 recruitment to membranes. Overall, these results indicate that the expression of Rab21 DN mutant affects EEA1 recruitment to membranes and this might result in the reduction in EE size.

### Rab5 mislocalizes to the Golgi apparatus and fails to recruit EEA1 on EEs in presence of Rab21 T31N

**FIGURE 2:**
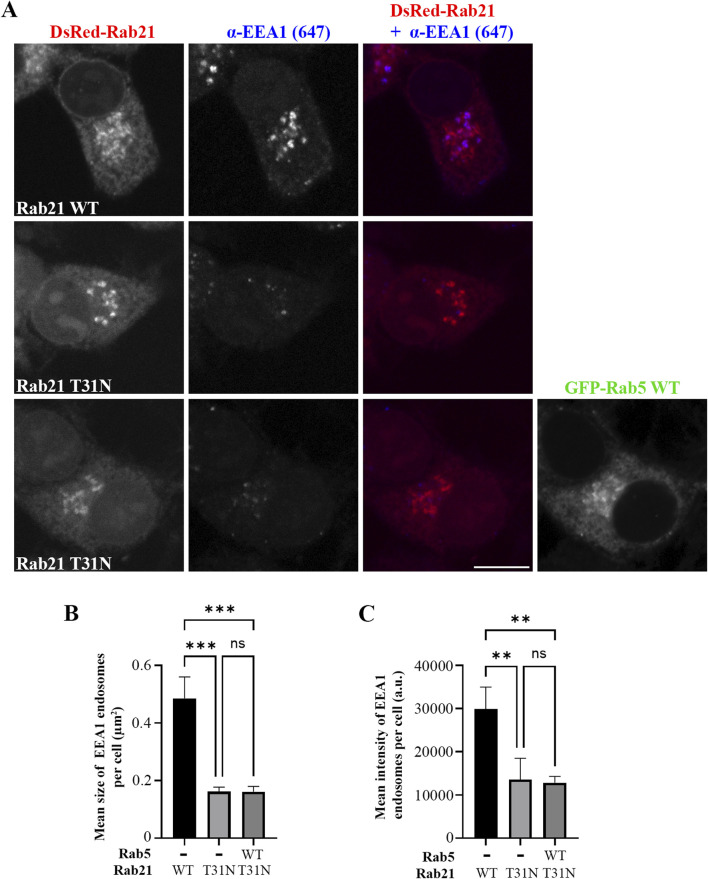
Rab5 overexpression does not rescue the alteration in EEs caused by the DN mutant of Rab21. **(A)** Representative images of N2a cells transfected with DsRed-Rab21 WT or T31N, or co-transfected with DsRedRab21 T31N and GFP-Rab5 WT and immunostained with an antibody against EEA1. Scale bar: 10 µm. Quantification of the mean size of EEA1-positive endosomes per cell **(B)** and of the mean intensity of EEA1-positive endosome per cell **(C)** in the cells transfected as in (a). The graphs represent the mean and standard deviation of three independent experiments. n ≥ 50 cells per condition. For statistical analysis One-way ANOVA followed by Tukey’s multiple comparation test was performed. **P < 0.01; ***P < 0.001.

**FIGURE 3:**
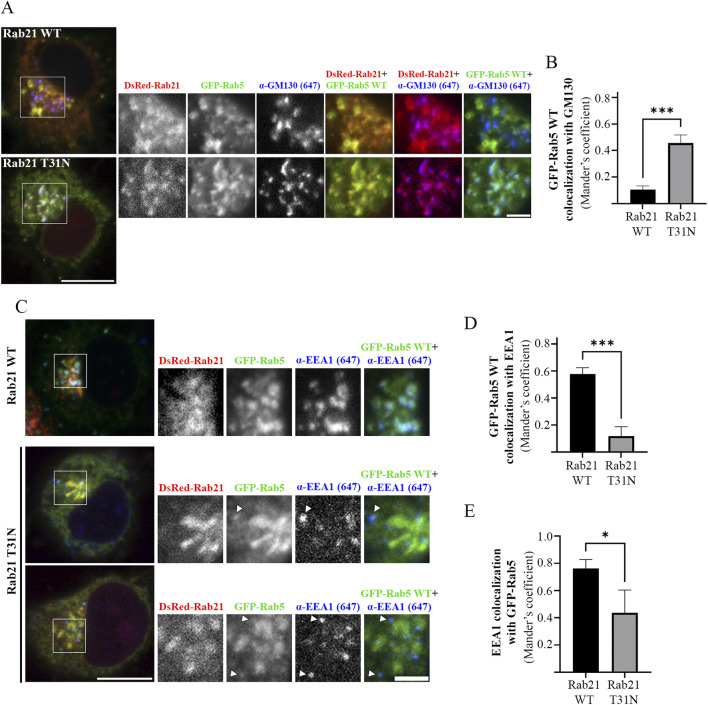
Rab5 mislocalizes to the Golgi apparatus and fails to recruit EEA1 on EEs in presence of Rab21 T31N. **(A)** Representative images of N2a cells co-transfected with GFP-Rab5 WT and either DsRed-Rab21 WT or T31N mutant and immunostained with antibodies against GM130 and GFP. Insets on the right show magnification of the boxed areas. Scale bar: 10 μm; inset: 3 µm. **(B)** Quantification of Mander’s colocalization coefficient between GFP-Rab5 WT and GM130 in N2a cells expressing DsRed-Rab21 WT or T31N. **(C)** Representative images of N2a cells co-transfected with GFP-Rab5 WT and either DsRed-Rab21 WT or T31N mutant and immunostained with antibodies against EEA1 and GFP. In DsRed-Rab21 T31N expressing cells EEA1 channel brightness has been increased to better visualize the vesicles. Arrowheads indicate EEA1-positive endosomes with no or weak GFP-Rab5 signal. Insets on the right show magnification of the boxed areas. Scale bar: 10 μm; inset: 3 µm. **(D)** Quantification of Mander’s colocalization coefficient between GFP-Rab5 WT and EEA1 in N2a cells expressing DsRed-Rab21 WT or T31N. **(E)** Quantification of Mander’s colocalization coefficient between EEA1 and GFP-Rab5 WT in N2a cells expressing DsRed-Rab21 WT or T31N. In b, d, and e, graphs show the mean and standard deviation of three independent experiments. n ≥ 65 cells per condition. For statistical analysis Student’s t-test was performed. *P < 0.05; ***P < 0.001.

As we determined that the expression of Rab21 DN mutant decreases the size of EEA1-positive endosomes and the recruitment of EEA1 to membrane, and as EEA1 recruitment to early endosomes is regulated by Rab5 (Callaghan et al., 1999a; Simonsen et al., 1998), we next investigated whether the overexpression of Rab5 is able to recover the defect caused by Rab21 DN mutant. Interestingly, the expression of GFP-Rab5 WT did not rescue the alteration in EEA1-positive endosomes caused by Rab21 DN mutant (Figure 2). In addition, we observed that GFP-Rab5 WT localization seems to be affected by the expression of Rab21 T31N. DsRed-Rab21 DN mutant is present, as previously described, on the Golgi complex (Simpson et al., 2004; Figure 3A). Surprisingly, GFP-Rab5 WT relocalized to the Golgi in presence of Rab21 DN mutant as revealed by the labelling with the Golgi marker GM130 (Figure 3A). Indeed, when expressing DsRed-Rab21 WT, colocalization of GFP-Rab5 WT with GM130 was very low (Mander’s coefficient 0.1). However, we measured a 4,8-fold increase in colocalization when expressing DsRed-Rab21 DN mutant (Figure 3B), confirming that the expression of Rab21 T31N mislocalizes Rab5 to the Golgi. Altered distribution of GFP-Rab5 WT was also observed in HeLa cells expressing DsRed-Rab21T31N, indicating that the effect is general and not cell line specific (Supplementary Figure S2A).

Since EEA1-positive endosomes are smaller when expressing Rab21 T31N compared to Rab21 WT, and GFP-Rab5 WT is mislocalized to the Golgi complex, we wondered if the colocalization between Rab5 and its effector EEA1 is affected by the expression of Rab21 DN mutant. As expected, in cells transfected with DsRed-Rab21 WT most of EEA1-positive endosomes colocalized with GFP-Rab5 WT (Mander’s coefficient 0.75), (Figures 3C,E). Also, a big percentage of GFP-Rab5 endosomes colocalized with EEA1 (Mander’s coefficient 0.57), (Figures 3C,D). However, in cells expressing DsRed-Rab21 DN mutant, a 80% reduction in the colocalization of GFP-Rab5 with EEA1 was measured (Figures 3C,D). This is in accordance with the mislocalization of GFP-Rab5 to the Golgi (Figures 3A,B). In addition, in the presence of Rab21 DN mutant, it was measured more than 40% reduction in colocalization of EEA1 with GFP-Rab5 indicating that some of the small EEA1 vesicles are not Rab5-positive (Figure 3C, arrows, and E). This effect was also observed in HeLa cells where EEA1-positive-endosomes with very weak or not detectable GFP-Rab5 signal are present (Supplementary Figure S2B, arrows).

Overall, these results indicate that the localization of EEA1 and Rab5 is altered upon the expression of Rab21 DN mutant. This defect is restricted to EEs because Rab11 and Lamp1-positive endosomes are not altered when expressing Rab21 DN, as shown by the lack of colocalization with GM130 (Supplementary Figure S3).

### Rab21 regulates endosomal size by interacting with and recruiting EEA1 to endosomal membranes

**FIGURE 4:**
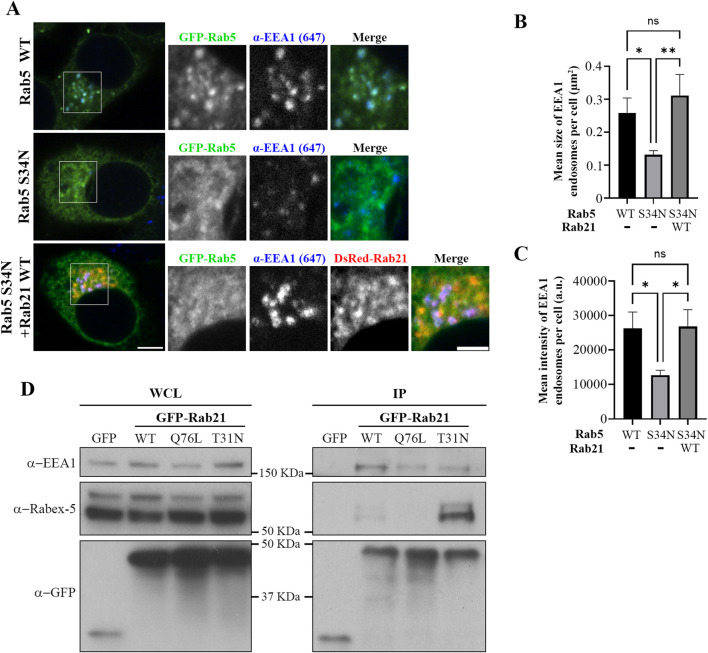
Rab21 interacts with EEA1 and rescues its recruitment to EEs in presence of the dominant negative mutant of Rab5. **(A)** Representative images of N2a cells transfected with either GFP-Rab5 WT or S34N, or co-transfected with GFP-Rab5 S34N and DsRed-Rab21 WT and immunostained with antibodies against EEA1 and GFP. Insets on the right show magnification of the boxed areas. Scale bar: 5 μm; inset 3 µm. Quantification of the mean size of EEA1-positive endosomes per cell **(B)** and of the mean intensity of EEA1-positive endosome per cell **(C)** in cells transfected as in (a). In b and c, the graphs represent the mean and standard deviation of three independent experiments. n ≥ 50 cells per condition. **(D)** N2a cells were transfected with either GFP, GFP-Rab21 WT, Q76L or T31N, lysed and subjected to IP with GFP magnetic agarose beads. Whole cell lysates (WCL) and immunoprecipitates (IP) were subjected to Western blot analysis with the indicated antibodies. For statistical analysis One-way ANOVA followed by Tukey’s multiple comparation test was performed. *P < 0.05; **P < 0.01.

EEA1 is recruited to the EEs by active Rab5 (Simonsen et al., 1998). Our results showed that the expression of Rab21 DN mutant affects the localization of both Rab5 WT and EEA1. Therefore, we wondered if this alteration of EEA1 localization can be explained only by the mislocalization of Rab5 or if also Rab21 actively contributes to facilitate the localization of EEA1. To investigate if Rab21 might have a role recruiting EEA1 independently of Rab5, we next explored if the expression of Rab21 WT rescues EEA1 recruitment to endosomes in the presence of Rab5 dominant negative mutant S34N, which is known to prevent EEA1 recruitment to EEs (Dinneen and Ceresa, 2004; Johns et al., 2009). To this end, we quantified the mean size and the mean intensity of EEA1-positive endosomes per cell in cells expressing GFP-Rab5 WT, GFP-Rab5 S34N, or co-expressing GFP-Rab5 S34N together with DsRed-Rab21 WT. As expected, GFP-Rab5 S34N presented a cytosolic distribution and its expression altered EEA1-positive endosomes, reducing their area and mean intensity (Figures 4A–C). Intriguingly, when DsRed-Rab21 WT was co-expressed together with GFP-Rab5 DN mutant, the mean area and the mean intensity of EEA1-positive endosomes per cell were restored (Figures 4A–C). This indicates that the expression of Rab21 WT compensates for the loss of function of Rab5 regarding EEA1 localization and endosomal size. In these cells, DsRed-Rab21 WT is still present on EEs together with EEA1 (Figure 4A; Supplementary Figure S4A), further suggesting that the localization of Rab21 is independent of Rab5 function.

**FIGURE 5:**
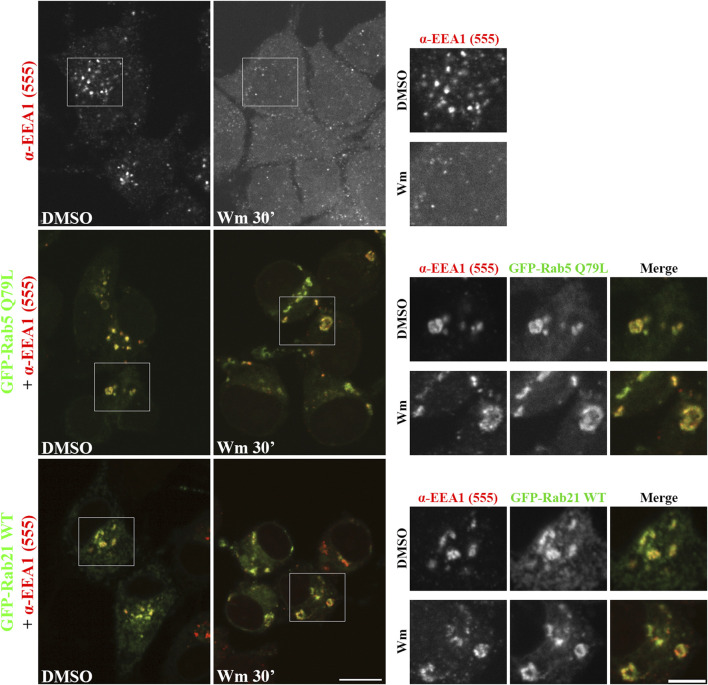
Rab21 recruits EEA1 to endosomes in PI3P depleted cells. Representative images of N2a cells transfected with either GFP-Rab5 Q79L or GFP-Rab21, or non-transfected. Cells were treated with either 10 µM wortmannin (Wm) or DMSO for 30 min, then fixed and stained with an antibody against EEA1. Insets on the right show magnification of the boxed areas. Scale bar: 10 μm; inset 5 µm.

The fact that the expression of Rab21 can rescue EEA1 mis-localization caused by Rab5 DN mutant confirms that Rab21 has a role controlling EEA1 localization independently of Rab5 and suggests that the function of Rab5 and Rab21 might be partially redundant regarding EEA1 recruitment. In agreement with this, we detected that Rab21 interacts with EEA1 by co-immunoprecipitation (Figure 4D). To further confirm the role of Rab21 in the recruitment of EEA1 to endosomes, we took advantage of the phosphatidylinositol 3-phosphate (PI3P)-kinase inhibitor wortmannin. This inhibitor abolishes the generation of PI3P inducing the detachment of EEA1 from EEs (Patki et al., 1998; Patki et al., 1997), and in line with that, in non-transfected cells EEA1 loses its characteristic early endosomal localization after a treatment of 30 min with 10 µM wortmannin (Figure 5). Consistently with previous work (Simonsen et al., 1998), the expression of Rab5 constitutively active (CA) mutant Q79L overcomes the effect of wortmannin and retains EEA1 on the endosomal membranes (Figure 5). Surprisingly, in GFP-Rab21 WT transfected cells, EEA1 is still present on Rab21-positive endosomes even after the treatment with wortmannin (Figure 5). In these conditions the Rab21 GEF, Rabex-5, is still present on Rab21-positive endosomes (Supplementary Figure S5A) supporting that Rabex-5 localization is not dependent on PI3P and that is able to activate Rab21 even in presence of wortmannin.

To further confirm that the Rab21-dependent recruitment of EEA1 to endosomes in presence of wortmannin is not due to the PI3P synthesized by the class II PI3K-C2α enzyme that is less sensitive to wortmannin (Wen et al., 2008), we co-expressed GFP-Rab21 or GFP-Rab5 CA together with the PI3P probe mCherry-2xFYVE. As expected, wortmannin treatment re-distributed mCherry-2xFYVE to the cytosol, although in some cells it was still present on vesicles (Supplementary Figure S5B). This is in agreement with previous work showing the existence of a pool of PI3P, synthesized by the less sensitive to wortmannin PI3K-C2α, in secretory vesicles (Wen et al., 2008). In accordance with this, in GFP-Rab21 or GFP-Rab5 CA expressing cells mCherry-2xFYVE was not present on EE-positive for Rab21 or Rab5 CA mutant (Supplementary Figure S5B).

Altogether, these results confirm the role of Rab21 in recruiting EEA1 to the endosomal membrane, further suggesting that this can occur independently of PI3P, and support a model in which Rab21 regulates endosomal size by facilitating the recruitment of EEA1 to the endosomes.

### Rab21 T31N mislocalizes Rab5 by competing for Rabex-5

Intriguingly, our results show that the expression of Rab21 DN mutant alters the localization of Rab5. Therefore, we wondered by which mechanism does Rab21 DN mutant disturb the endosomal localization of Rab5. GEFs are major determinants for Rab localization (Blümer et al., 2013; Wu et al., 2010). It is well established that the early endosomal GEF Rabex-5 interacts and has *in vitro* activity towards Rab5 and also regulates its localization (Blümer et al., 2013; Delprato et al., 2004). Moreover, the overexpression of Rabex-5 restores the endosomal localization of Rab5 DN (Mattera and Bonifacino, 2008; Zhu et al., 2007; Supplementary Figure S4B). Rabex-5 has also *in vitro* activity towards Rab21 and interacts with it (Delprato et al., 2004; Delprato and Lambright, 2007; Mori et al., 2013). In agreement with this, we confirmed the interaction between Rab21 and Rabex-5 by co-immunoprecipitation (Figure 4D). This interaction is stronger with the DN mutant of Rab21 than with the WT, as has been detected before in a yeast two hybrids assay and as is expected for GEFs and their target Rabs (Mori et al., 2013; Delprato and Lambright, 2007). Interestingly, we show that the overexpression of Rabex-5 restores the endosomal localization of Rab21 DN as it does with Rab5 DN (Supplementary Figure S4C).

**FIGURE 6:**
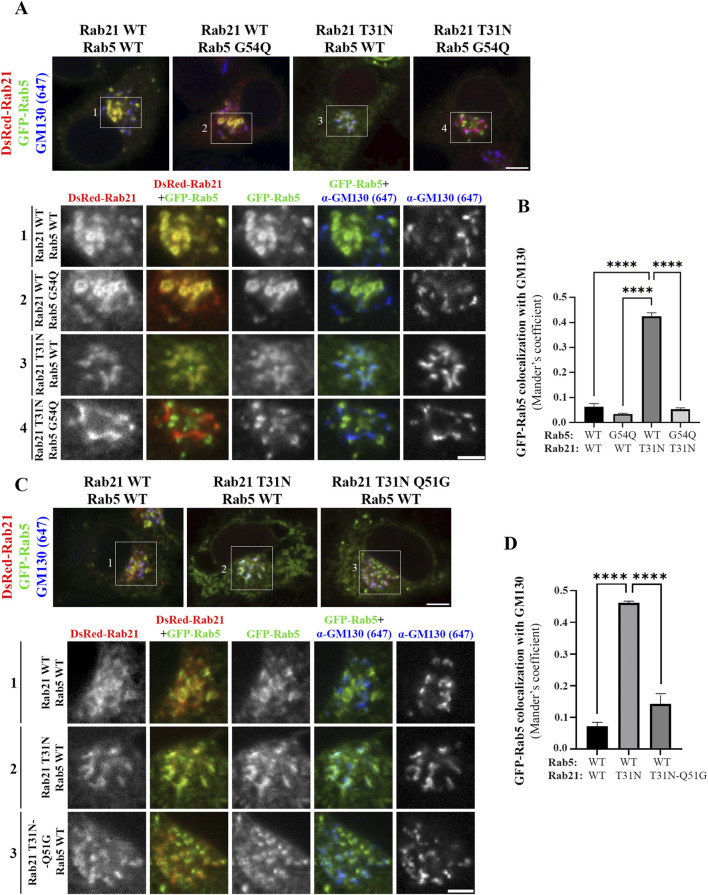
Rabex-5 increased activity towards Rab5 or reduced binding to Rab21 T31N rescue Rab5 mislocalization induced by Rab21 T31N. **(A)** Representative images of N2a cells co-transfected with the indicated constructs and immunostained with antibodies against GM130 and GFP. Insets below show magnification of the boxed areas. Scale bar: 5 μm; inset: 3 µm. **(B)** Quantification of Mander’s colocalization coefficient between GFP-Rab5 and GM130 in cells transfected as in a. **(C)** Representative images of N2a cells co-transfected with the indicated constructs and immunostained with antibodies against GM130 and GFP. Insets below show magnification of the boxed areas. Scale bar: 5 μm; inset: 3 µm. **(D)** Quantification of Mander’s colocalization coefficient between GFP-Rab5 and GM130 in cells transfected as in c. In b and d, graphs show the mean and standard deviation of three independent experiments. n ≥ 45 or 53 cells in b and d, respectively. For statistical analysis One-way ANOVA followed by Tukey’s multiple comparation test was performed. ****P < 0,0001.

Therefore, as Rabex-5 acts as a GEF for both Rab GTPases it could be an important point of cross regulation between Rab21 and Rab5. If Rab21 and Rab5 compete for their binding to Rabex-5, it might explain why the expression of the DN mutant of Rab21, that binds more to Rabex-5 than the WT version, causes the mislocalization of Rab5. In this scenario, most of Rabex-5 might be bound to Rab21 DN mutant depleting the pool of free Rabex-5 necessary to activate Rab5. To test this hypothesis, we took advantage of a point mutation previously reported in human Rab5 (G55Q) to produce a 12-fold increase in the *in vitro* activity of Rabex-5 towards Rab5 (Delprato et al., 2004). Thus, we made the equivalent point mutation in the canine GFP-Rab5 sequence (G54Q) to assess if this mutation, that leads to a more efficient activation of Rab5 by Rabex-5, restores the normal localization of this small GTPase when co-expressed with Rab21 DN mutant. GFP-Rab5 G54Q is present on Rab21-positive endosomes when co-expressed with dsRed-Rab21 WT, and does not colocalize with the Golgi marker GM130, similar to GFP-Rab5 WT (Figures 6A,B). Unlike GFP-Rab5 WT, GFP-Rab5 G54Q when co-expressed with DsRed-Rab21 DN mutant, still presents a vesicular distribution showing no colocalization with the Golgi apparatus (Figures 6A,B). This indicates that distinct to Rab5 WT, the localization of Rab5 G54Q is not affected by the expression of Rab21 DN mutant. This suggests that enhancing Rab5 activation by Rabex-5 is enough to restore the normal localization of Rab5 in presence of Rab21 DN mutant.

A point mutation in human Rab21 (Q53G) has previously been reported to produce a 60-fold decrease in the *in vitro* activity of Rabex-5 towards Rab21 (Delprato et al., 2004). Thus, to complement the previous experiment, we made an equivalent point mutation in Rab21 DN mouse sequence (Q51G). Co-immunoprecipitation analysis showed that the Q51G point mutation prevents the interaction between Rab21 DN and Rabex-5 *in vivo* explaining why Rabex-5 has reduced *in vitro* activity towards it (Delprato et al., 2004; Supplementary Figure S6A). Consequently, Rab21 DN Q51G should be a weaker target for Rabex-5, leaving Rabex-5 available to activate Rab5. DsRed-Rab21 DN Q51G presents a Golgi localization and a more cytoplasmic distribution than DsRed-Rab21 WT in accordance with its DN condition (Figure 6C). Co-expression of DsRed-Rab21 DN Q51G with GFP-Rab5 WT does not induce the mislocalization of Rab5 to the Golgi apparatus observed in the presence of DsRed-Rab21 DN (Figures 6C,D). These results support a model in which Rab5 and Rab21 compete for the binding and activation by Rabex-5.

**FIGURE 7:**
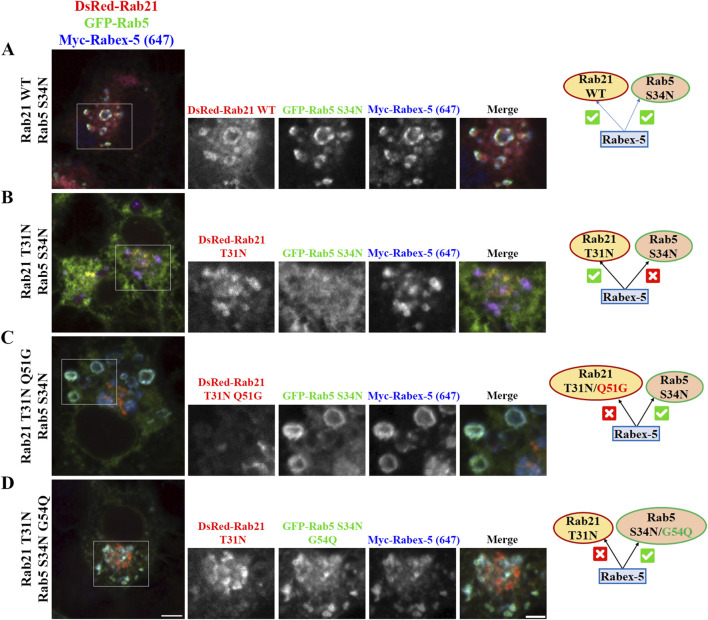
Rabex-5 overexpression rescues the endosomal localization of Rab5 S34N in Rab21 T31N expressing cells when Rabex-5 activity towards Rab5 is enhanced or binding to Rab21 T31N is prevented. **(A–D)** Representative images of N2a cells co-transfected with the indicated constructs and immunostained with antibodies against Myc and GFP. Insets on the right show magnification of the boxed areas. Scale bar: 5 μm; inset: 3 µm. Schematic representations of the results are shown. A green symbol indicates rescue of endosomal localization by Rabex-5 overexpression and a red symbol indicates prevented endosomal localization.

To further confirm this model and better understand the relationship between Rabex-5, Rab21 and Rab5, we took advantage of the ability of Rabex-5 to restore the endosomal localization of Rab21 T31N and Rab5 S34N when it is overexpressed, as shown before (Mattera and Bonifacino, 2008; Zhu et al., 2007; Supplementary Figures S4B,C). When DsRed-Rab21 WT is expressed together with GFP-Rab5 S34N and Myc-Rabex-5, Rab5 S34N is present on endosomes positive for both Rab21 and Rabex-5 (Figure Figure7A). This is consistent with the ability of overexpressed Rabex-5 to restore the endosomal localization of Rab5 S34N (Mattera and Bonifacino, 2008; Zhu et al., 2007; Supplementary Figure S4B). However, Rab5 S34N does not localize to endosomes when it is overexpressed together with Myc-Rabex-5 and DsRed-Rab21 T31N (Figure 7B). On the contrary, Rab21 T31N partially relocalizes to endosomes when Myc-Rabex-5 is expressed (Figure 7B). This result further supports the model in which Rab21 and Rab5 compete for Rabex-5 and suggests higher affinity of Rab21 than Rab5 for this GEF since Rab21 T31N but not Rab5 S34N relocates to endosomes when they are co-expressed together with Rabex-5. This might be supported structurally by different interaction properties of these GTPases with Rabex-5 around the switch I region. In humans, Rab21 Gln53 stabilizes the open switch conformation through polar interactions with the backbone of Ser55 and Phe56. This allows Ser55 and Ala54 to bind to Ala310 in Rabex-5 by hydrogen bonds (Delprato and Lambright, 2007; Supplementary Figure S6B). AlphaFold 3 predictions (Abramson et al., 2024) for Rab5-Rabex-5 interaction shows that Gly54 in Rab5 does not interact with other residues and there is only one hydrogen bond between Rab5 Ala55 and Rabex-5 Ala310 in this region (Supplementary Figure S6C). Similarly to the predicted structure of Rab5-Rabex-5 complex, AlphaFold 3 predictions with human Rab21 Q53G and Rabex-5 show that Gly53 is unable to interact with Ser55 and Phe56 and therefore Ser55 loses its binding to Ala310 in Rabex-5 (Supplementary Figure S6B). In addition, it might affect the conformation of the switch I region (Delprato and Lambright, 2007). Ser55 is an important determinant for the specificity of Rabex-5 binding to Rab21 (Delprato et al., 2004) and the conformation of the switch I region is an important general determinant for the recognition of Rab GTPases by GEFs (Stein et al., 2012). This is in line with our CoIP result showing no interaction between murine Rab21T31N Q51G mutant and Rabex-5 (Supplementary Figure S6A). On the other hand, an AlphaFold 3 simulation using Rab5 G54Q mutant shows that, similarly to Rab21 WT, Gln54 establishes a polar interaction with Phe57 (Supplementary Figure S6C). This allows Ile53 to interact with Ala310 in Rabex-5 strengthening the interaction with the GEF. This is in accordance with the localization of Rab5 G54Q not being affected by the expression of Rab21 DN (Figures 6A,B).

To finally validate the model of competition between Rab21 and Rab5 for Rabex-5, we expressed GFP-Rab5 S34N and Myc-Rabex-5 together with DsRed-Rab21 DN Q51G, the mutant that does not interact with Rabex-5 (Supplementary Figure S6A). Our results show that Rab5 S34N, but not Rab21 DN Q51G, is present on endosomes positive for Rabex-5 (Figure 7C). The fact that the overexpression of Rabex-5 is not able to recruit Rab21 DN Q51G to endosomes is in agreement with the low ability of Rab21 DN Q51G to interact with Rabex-5 (Supplementary Figure S6A). As Rabex-5 is not bound to Rab21 DN Q51G, it can instead recruit Rab5 S34N to endosomes. Ultimately, we analyzed the effect of the overexpression of Rabex-5 while co-expressing GFP-Rab5 DN G54Q (which should be a better target of Rabex-5 (Delprato et al., 2004; Supplementary Figure S6C)) together with DsRed-Rab21 DN. In line with our model, Rabex-5 induces the endosomal localization of GFP-Rab5 DN G54Q but not of DsRed-Rab21 DN (Figure 7D). This result confirms the model in which Rab5 and Rab21 compete for the binding to Rabex-5 and suggests that Rab21 is a better interactor than Rab5.

Overall, our results indicate that Rab21 DN competes with Rab5 for the binding to Rabex-5 and prevents the activation of Rab5 by this GEF. The lack of efficient activation leads to the mislocalization of Rab5. Our data confirm the importance of Rabex-5 for regulating the localization and function of these two GTPases and reveal that Rabex-5 is a critical player in the cross-regulation between Rab21 and Rab5 at EEs.

## Discussion

### Rab21 regulates EEA1 recruitment to EEs and EE formation

Our data indicates that EEs are affected upon the expression of Rab21 DN mutant. EEA1-positive endosomes are smaller and EEA1 recruitment to the EE membrane is reduced when expressing Rab21 DN. In addition, Rab5 relocalizes to the Golgi apparatus in presence of Rab21 DN. Defective recruitment of EEA1 and mislocalization of Rab5 explain the reduced size of EEs since EEA1 and Rab5 are needed for endosome fusion and formation (Christoforidis et al., 1999; Callaghan et al., 1999a; Simonsen et al., 1998; Lawe et al., 2002; McBride et al., 1999; Murray et al., 2016). The alteration in EEA1 distribution might be a consequence of Rab5 mislocalization as Rab5 facilitates EEA1 recruitment to EEs and it is needed for its tethering function (Callaghan et al., 1999a; Simonsen et al., 1998; Lawe et al., 2002). However, our data also support an active role of Rab21 regulating EEA1 localization and EE formation. First, we show that Rab21 interacts with EEA1 by coimmunoprecipitation. This is also in accordance with previous results that detected EEA1 in Apex2-mediated Rab21 proximity labeling (Del Olmo et al., 2019b). Second, the overexpression of Rab21 WT rescues the defects of EEA1 localization and EE size produced by the expression of Rab5 DN mutant. This means that Rab21 can perform its function at EEs independently of Rab5 activity. Lastly, the overexpression of Rab21 WT overcomes the reduction in EEA1-positive endosomes caused by wortmannin, a drug which depletes PI3P, needed for EEA1 binding to EEs (Patki et al., 1998; 1997). The expression of Rab5 CA mutant has been reported to similarly rescue EEA1 localization after wortmannin treatment, and it has been proposed that Rab5 CA stabilizes EEA1 at the membrane through protein-protein interaction (Simonsen et al., 1998; Jones et al., 1998; Li et al., 1995). Rab21 might stabilize EEA1 at the endosome membrane through a similar mechanism. This might be supported by the interaction detected between EEA1 and Rab21 (Figure 4D). Altogether, our data indicates that Rab21 is also involved in EEA1 recruitment and EE formation, suggesting that Rab21 and Rab5 recruit EEA1 using parallel routes. Recently, it has been shown that Rab21 participates in caveolin-mediated endocytic transport and in integrin endocytosis through a clathrin independent pathway (Moreno-Layseca et al., 2021; Shikanai et al., 2023). So, it is tempting to speculate that Rab21-mediated recruitment of EEA1 might specifically regulate the fusion of endosomes in these pathways.

### Rab21 and Rab5 compete for Rabex-5 activation

Our results show that the expression of Rab21 DN affects Rab5 localization. Furthermore, our data supports a model in which this alteration is caused by the competition between Rab5 and Rab21 for the common GEF Rabex-5. It has been proposed that the mechanism by which a dominant negative mutant of a GTPase inhibits the function of the endogenous protein is by interacting with GEFs and sequestering them, thus they cannot activate the WT protein (Feig, 1999; Ridley, 2000). According to this and to previous yeast two-hybrid analysis (Mori et al., 2013), we detected more binding of Rabex-5 to Rab21 DN than to the WT protein by coimmunoprecipitation. This might induce a depletion in the pool of Rabex-5 available to activate Rab5. Indeed, when the interaction of Rab21 DN with Rabex-5 is reduced by introducing the point mutation Q51G, the localization of Rab5 is not affected. Furthermore, when the exchange activity of Rabex-5 over Rab5 is increased by expressing Rab5 G54Q (Delprato et al., 2004), this mutant retains endosomal localization even in the presence of Rab21 DN. In addition, the competition of Rab5 and Rab21 for Rabex-5 activation is also supported by the experiments performed overexpressing Rabex-5. The overexpression of Rabex-5, which is known to restore the endosomal localization of Rab5 S34N (Mattera and Bonifacino, 2008; Zhu et al., 2007), is not sufficient to induce the endosomal localization of Rab5 DN when it is expressed together with Rab21 DN. Moreover, in presence of Rab21 DN mutant containing the point mutation Q51G that reduces the *in vitro* exchange activity by rabex-5 and the binding to this GEF (Delprato et al., 2004; Supplementary Figure S6A), the overexpression of Rabex-5 relocates Rab5 DN to endosomes, consistent with the fact that Rab5 DN mutant has higher affinity for Rabex-5 than Rab21 DN Q51G (Supplementary Figure S6). Accordingly, the overexpression of Rabex-5 recruits Rab5 DN G54Q to endosomes even in the presence of Rab21 DN, as the point mutation G54Q might increase Rab5 affinity for Rabex-5 (Delprato et al., 2004; Supplementary Figure S6C). The competition of these two GTPases for the binding to Rabex-5 is also supported by the fact that they interact with the same region present in the Vps9 domain of Rabex-5 (Delprato et al., 2004; Delprato and Lambright, 2007).

In addition, our results and AlphaFold 3 predictions suggest that Rab21 has more affinity for Rabex-5 *in vivo* than Rab5. Supporting this, the expression of Rab5 DN does not affect Rab21 localization, while Rab21 DN prevents Rab5 recruitment to endosomes. Furthermore, the overexpression of Rabex-5 rescues, at least partially, the endosomal localization of Rab21 DN but not of Rab5 DN when the three proteins are expressed together. Additionally, the higher affinity of Rab21 for Rabex-5 would explain why the overexpression of Rab5 WT is not able to rescue the alteration of EEs caused by Rab21 DN. In this situation, Rab5 is mislocalized and may not be activated to perform its function at EEs because Rab21 DN sequesters its GEF Rabex-5. Previous works have established that the *in vitro* activity of Rabex-5 over Rab21 is similar to the one for Rab5 (Delprato et al., 2004; Delprato and Lambright, 2007). This further supports that the preference of Rabex-5 for Rab21 that we observe in the cell is due to a difference in interaction affinity rather than in a difference in the catalytic activity. A previous work identified that Rab21 and Rab5 have different specificity determinants for Rabex-5 exchange activity according to their structure (Delprato et al., 2004). While the specificity for Rab5 is based on the cumulative contribution of multiple weak determinants, the specificity for Rab21 is highly dependent on the residue Q53 of human protein (equivalent to Q51 of the murine protein). In line with this, our results show that amino acid Q51 in murine Rab21 is an important residue for the interaction with Rabex-5. In addition, the reciprocal change G54Q in Rab5 sequence makes Rab5 a better target for Rabex-5, enabling it to take over Rab21 in the competition for the activation by this GEF. This is also supported by AlphaFold 3 simulations (Supplementary Figures S6A,B). Therefore, the presence of Q in this position of the protein is an important factor for the different binding properties of these GTPases for Rabex-5.

Our results show that the expression of Rab21 DN affects Rab5 localization presenting a more diffuse distribution in the cell and localizing to the Golgi. Due to the competition between Rab21 and Rab5 for the binding to Rabex-5, it is not surprising that the endosomal localization of Rab5 is affected in cells expressing Rab21 DN. Rab21 DN depletes the pool of available Rabex-5 by binding to it, so Rab5 cannot be activated and does not bind to the membrane of the endosomes. The fact that Rab5 relocalizes to the Golgi complex when Rab21 DN mutant is expressed might also be related to its low activation state. Indeed, dominant negative mutants of some members of the Rab5 subfamily, including Rab21 and Rab22 DN, have been reported to localize to the Golgi complex (Simpson et al., 2004; Kauppi et al., 2002 and our results). Rab5 S34N has been previously observed to present a perinuclear staining in addition to a cytoplasmic distribution (Zhu et al., 2007), and in line with that, we detected a partial colocalization of Rab5 S34N perinuclear structures with the Golgi (Supplementary Figure S7). This is also in agreement with the localization of Rab5 A56D/Y82A, a mutant with reduced nucleotide exchange susceptibility for Rabex-5, that localizes at perinuclear compartments that resemble Golgi cisternae and not at endosomes (Blümer et al., 2013). Altogether these data support that the mislocalization of Rab5 to the Golgi is a consequence of its inactive state due to the sequestration of Rabex-5 by Rab21 DN.

In conclusion, our work defines the role of Rab21 in regulating EE formation through EEA1 recruitment and describes a model in which Rab21 and Rab5 compete for the binding to Rabex-5, which is a new mechanism of regulation between these GTPases. This implies that activation by Rabex-5 is a critical point of coordination between these GTPases at the EEs.

## Material and methods

### Cell culture

Neuro2A (N2a) and HeLa cells were grown in DMEM (Biowest) supplemented with 10% FCS, 2 mM L-glutamine, 100 U/mL penicillin, and 100 μg/mL streptomycin kept at 37°C and 5% CO_2_.

### Antibodies and constructs

Primary antibodies used were: Anti-EEA1 from Santa Cruz (sc-137130) was diluted 1:1,000 for Western blot and 1:50 for immunostaining (IF). Anti-tubulin Sigma (T9026) was used at 1:100,000 dilution for Western blot. Anti-Sortilin from Abcam (Ab16640) was used at 1:2,000 dilution for Western blot. Anti-GFP from Abcam (Ab6556) was used at 1:2,000 dilution for Western blot and 1:200 for IF. Anti-Myc from Abcam (Ab32) was used at 1:100 dilution for IF. Anti-GM130 from BD transduction laboratories (610822) was used at 1:100 dilution for IF. Anti-Lamp1 from Santa Cruz (Sc-19992) was used at 1:200 dilution for IF. Anti-Rabex5 from Proteintech (12735-1-AP) was used at 1:1,000 dilution for Western blot and 1:50 dilution for IF. Anti-HA from abcam (Ab9110) was used at 1:100 dilution for IF.

For immunofluorescence experiments, Alexa Fluor secondary antibodies (Invitrogen) were used at dilution 1:200. Secondary antibodies conjugated to HRP (GE Healthcare) were diluted 1:5,000 for immunoblotting studies.

The following constructs were gifts from Johanna Ivaska: pEGFP-Rab21, (Addgene plasmid # 83421; http://n2t.net/addgene:83421; RRID: Addgene_83421; Pellinen et al., 2006), pEGFP-Rab21-T31N (DN) (Addgene plasmid # 83423; http://n2t.net/addgene:83423; RRID: Addgene_83423; Pellinen et al., 2006), pEGFP-Rab21-Q76L (CA) (Addgene plasmid # 83422; http://n2t.net/addgene:83422; RRID: Addgene_83422; Pellinen et al., 2006) and dsRedm-Rab21 (Addgene plasmid # 83425; http://n2t.net/addgene:83425; RRID:Addgene_83425; Pellinen et al., 2008). HA-Rab11-WT was a gift from Ken-Ichi Takemaru (Addgene plasmid # 101047; http://n2t.net/addgene:101047; RRID:Addgene_101047; Burke et al., 2014). pCI-neo-Myc-Rabex-5 was a gift from Juan Bonifacino (Addgene plasmid # 196937; http://n2t.net/addgene:196937; RRID:Addgene_196937; Mattera et al., 2006). pEGFPC1-Rab5 WT was a kind gift of Cecilia Bucci (University of Salento, Italy). GFP-Rab5DN(S34N) was a gift from Sergio Grinstein (Addgene plasmid # 35141; http://n2t.net/addgene:35141; RRID:Addgene_35141; Bohdanowicz et al., 2012) and EGFP-Rab5A Q79L was a gift from Qing Zhong (Addgene plasmid # 28046; http://n2t.net/addgene:28046; RRID:Addgene_28046; Sun et al., 2010). pmCherry-2xFYVE was a kind gift of Kay Oliver Schink (University of Oslo) (Sneeggen et al., 2019).

To make the DsRedm-Rab21 T31N plasmid the sequence of WT Rab21 was substituted in the dsRedm-Rab21 plasmid by the sequence of Rab21 T31N from the pEGFP-Rab21 T31N plasmid by using HindIII and XbaI restriction sites. DsRedm-Rab21 T31N with the point mutation Q51G, and pEGFPC1-Rab5 WT and GFP-Rab5DN(S34N) with the point mutation G54Q were obtained using the Quick Change II XL Site-Directed Mutagenesis Kit from Agilent Technologies according to manufacturer’s instructions. The following primers from Eurofins Genomics were used: Rab21Q51G-Rv 5′-CTTTGTTAAGAAAGATGCCCCCAGGGTGGTGATGTGCTTG-3′ and Rab21Q51G-Fw 5′-CAAGCACATCACCACCCTGGGGGCATCTTTCTTAACAAAG-3'; Rab5G54Q-Rv 5′-GTTTGGGTTAGAAAAGCAGCCTGTATGGTACTCTCTTGAAATTCATGAAATTGG-3′ and Rab5G54Q-Fw 5′-CCAATTTCATGAATTTCAAGAGAGTACCATACAGGCTGCTTTTCTAACCCAAAC-3'. The resulting plasmids were verified by sequencing.

### Transfection

N2a cells were transiently transfected using Lipofectamine 2000 (Life Technologies) following the manufacturer’s instructions. HeLa cells were transiently transfected using FuGENE (Promega) following the manufacturer’s instructions. Cells were transfected at 60%–70% confluence for 16–24 h before further execution of experiments.

### Western blotting

N2a cells were lysed in lysis buffer (125 mM K-acetate, 25 mM Hepes, 5 mM EGTA, and 2.5 mM Mg-acetate, pH 7.2) complemented with 0.5% NP-40, protease inhibitor cocktail (Roche), and DTT (Sigma-Aldrich). Lysates were subjected to centrifugation at 13,000 x g. Supernatants were diluted in 2X Laemmli sample buffer, subjected to SDS-PAGE and blotted onto polyvinylidene fluoride membranes (Millipore). The membranes were incubated overnight at 4°C with primary antibodies diluted in 2% blotting grade nonfat dry milk (Bio-Rad), followed by 1 h incubation at room temperature with secondary antibodies conjugated to HRP (Cytiva). Either the Amersham ECL Prime Western blotting Detection Reagent (Cytiva) or the SuperSignal West Femto Maximum Sensitivity Substrate (Thermo Scientific) were used for chemiluminescence detection. The chemiluminescent signal was detected on films (Amersham^TM^ Hyperfilm^TM^ ECL, Cytiva).

### Co-immunoprecipitation

For co-immunoprecipitation (co-IP) experiments, GFP-Trap_MA magnetic agarose beads (Chromotek) were used according to the manufacturer’s instructions. Briefly, cells were transfected with GFP-fusion proteins, lysed in lysis buffer (10 mM Tris-HCl, pH 7.5, 150 mM NaCl, 0.5 mM EDTA, 0.2% NP-40, protease inhibitor cocktail (Roche) and 1 mM PMSF) and subjected to centrifugation at 13,000 x g for 10 min. Supernatant was diluted 1:2 with washing buffer (10 mM Tris-HCl, pH 7.5, 150 mM NaCl, 0.5 mM EDTA, protease inhibitor cocktail (Roche) and 1 mM PMSF) and incubated with control magnetic agarose beads for 15 min at 4°C. The pre-cleared supernatant was incubated for 1 h at 4°C with magnetic agarose beads coupled to antibody against GFP for co-IP. After three steps of washing, immunoprecipitated samples and total lysates were loaded on SDS-PAGE gels and subjected to Western blotting analysis.

### Subcellular fractionation

N2a cells grown in 10 cm dishes were washed with 10 mL 1X PBS followed by a wash with 1 mL of homogenization buffer (8.5% sucrose, 50 mM HEPES, 10 mM KCl, 3 mM EGTA, pH = 7.3) with protease inhibitors (Roche). Cells were scraped and collected in 500 µL of homogenization buffer. A 25 G syringe ¾ needle was used to mechanically lyse the cells, before centrifugation at 3,000 x g for 10 min at 4°C to pellet the nuclei. Protein concentration of supernatant was measured by using NanoDrop 2000 (Thermo Scientific). Protein amount was equalized in 400 µL of final volume and the samples were subjected to ultracentrifugation at 100,000 x g for 1 h at 4°C using a Sorvall MTX150 Micro-ultracentrifuge (Thermo Scientific) equipped with a S55-A2 rotor. After ultracentrifugation, supernatant (i.e., cytosol) and pellet (i.e., membranes) were carefully separated and subjected to Western blot analysis. ImageJ was used to quantify the protein signal from the membrane and cytosolic fractions. For each condition, the ratio between the levels of protein in the membrane and cytosolic fractions was calculated.

### Immunofluorescence and live cell imaging

For immunofluorescence, cells were grown on glass coverslips, washed with 1X PBS, permeabilized by using 0.25% saponin in 1X PBS for 2 minutes (Sigma-Aldrich), fixed with 3% PFA for 20 min, quenched using 50 mM NH_4_Cl for 10 min and washed in 0.25% saponin in 1X PBS. Cells were incubated with primary antibodies at room temperature for 40 min, washed with 0.25% saponin in 1X PBS three times, incubated with secondary antibodies at room temperature for 20 min, washed again with 0.25% saponin in 1X PBS and mounted with Mowiol.

For experiments with wortmannin treatment, cells were incubated with 10 µM of Wortmannin (Sigma) or DMSO 30 min before proceeding with the fixation.

For live-cell imaging, cells were seeded on MatTek glass-bottom dishes. During imaging, the cells were kept at 37°C and 5% CO_2_.

For live-cell imaging an Olympus SpinSR SoRa spinning disk confocal with a 60X Plan Apo 1.42 NA oil objective was used. The rest of the images were acquired either on a Zeiss LSM880 Fast AiryScan confocal microscope with a C Plan Apo 63×/1.4NA oil objective or with an Andor Dragonfly spinning disk microscope equipped with a 60× Apo oil objective, NA 1.4.

### Image processing and analysis

Image processing and analysis were performed using ImageJ/Fiji software (National Institutes of Health). The JACoP plugin (Bolte and Cordelieres, 2006) was used to analyze protein colocalization by calculating the Mander’s colocalization coefficient, after applying the threshold for each channel. To analyze the endosomal size and mean intensity a mask was created by subtracting the background (rolling ball radius = 25) and filtering the image. Threshold was applied in the resulting image to create a binary image. Then the mean size of the endosomes was calculated by using the analyze particles function excluding particles smaller than 3 pixels. From the binary image a selection of the endosomes was created and exported to the original image to measure the mean intensity.

### Statistical analysis

Evaluation of statistical differences was done by using Graphpad Prism software. A Student’s unpaired t-test was performed when comparing two samples. One-way ANOVA followed by Tukey’s multiple comparation test was performed when comparing more than two samples.

## Data Availability

The raw data supporting the conclusions of this article will be made available by the authors, without undue reservation.

## Supplementary material

SUPPLEMENTARY FIGURE S1 The dominant negative mutant of Rab21 affects EEs in HeLa cells. Representative images of HeLa cells transfected with DsRed-Rab21 WT or DsRed-Rab21 T31N and immunostained with an antibody against EEA1. Insets on the right show magnification of the boxed areas. Scale bar: 10 µm; insets: 5 µm.

SUPPLEMENTARY FIGURE S2 Rab5 mislocalizes to the Golgi complex in presence of Rab21 T31N in HeLa cells. **(a)** Representative images of HeLa cells co-transfected with GFP-Rab5 WT and either DsRed-Rab21 WT or DsRed-Rab21 T31N and immunostained with antibodies against GM130 and GFP. Insets below show magnification of the boxed areas. **(b)** Representative images of HeLa cells co-transfected with GFP-Rab5 WT and either DsRed-Rab21 WT or DsRed-Rab21 T31N and immunostained with antibodies against EEA1 and GFP. Insets below show magnification of the boxed areas. Arrowheads indicate EEA1 endosomes with no or weak GFP-Rab5 signal. Scale bars: 10 µm; insets: 5 µm.

SUPPLEMENTARY FIGURE S3 Rab11 and Lamp1 do not mislocalize to the Golgi complex in the presence of Rab21 T31N. **(a)** Representative images of N2a cells co-transfected with HA-Rab11 and either DsRed-Rab21 WT or T31N and immunostained with antibodies against GM130 and HA. Insets on the right show magnification of the boxed areas. **(b)** Quantification of Mander’s colocalization coefficient between HA-Rab11 and GM130 in cells transfected as indicated in a. n ≥ 15 cells per condition. **(c)** Representative images of N2a cells transfected with either GFP-Rab21 WT or T31N and immunostained with antibodies against GM130 and Lamp1. Insets on the right show magnification of the boxed areas. In **(a,c)** scale bars: 5 µm; insets: 3 µm **(d)** Quantification of Mander’s colocalization coefficient between Lamp1 and GM130 in cells transfected with either GFP-Rab21 WT or T31N. n ≥ 17 cells per condition. In **(b,d)**, graphs represent the mean and standard deviation of one experiment.

SUPPLEMENTARY FIGURE S4 Rab21 remains at EEs in the presence of Rab5 S34N. Overexpression of Rabex-5 induces endosomal recruitment of Rab5 and Rab21 DN mutants. **(a)** Representative images of N2a cells co-transfected with DsRed-Rab21 and either GFP-Rab5 WT or S34N and immunostained with antibodies against EEA1 and GFP. Insets on the right show magnification of the boxed areas. Scale bar: 5 µm; insets: 3 µm. **(b)** Representative images of N2a cells transfected with GFP-Rab5 S34N or co-transfected with GFP-Rab5 S34N and Myc-Rabex-5 and immunostained with antibodies against Myc and GFP. Insets below show magnification of the boxed areas. **(c)** Representative images of N2a cells transfected with GFP-Rab21 T31N or co-transfected with GFP-Rab21 T31N and Myc-Rabex-5 and immunostained with antibodies against Myc and GM130. Insets on the right show magnification of the boxed areas. In **(b,c)** scale bars: 10 µm; insets: 5 µm.

SUPPLEMENTARY FIGURE S5 Wortmannin treatment does not affect Rabex-5 localization and retains Rab21 or Rab5 CA to endosomes independently of the presence of PI3P **(a)** Representative images of N2a cells transfected with either Myc-Rabex-5, Myc-Rabex-5 and GFP-Rab21 or not transfected. Cells were treated with either 10 µM wortmannin (Wm) or DMSO for 30 min, then fixed and immunostained with anti-EEA1 or with anti-Rabex-5 antibody. Insets on the right show magnification of the boxed areas. Scale bar: 10 µm; insets: 5 µm. **(b)** Representative images of N2a cells transfected with the indicated constructs. Cells were treated with either 10 µM wortmannin (Wm) or DMSO for 30 min and then visualized live. Insets on the bottom show magnification of the boxed areas. Scale bar: 10 µm; insets: 5 µm.

SUPPLEMENTARY FIGURE S6 Rab21 T31N Q51G does not interact with Rabex-5 and structure predictions of Rabex-5 in complex with nucleotide-free Rab21 WT or Q53G mutant, or with nucleotide-free Rab5 WT or G54Q mutant. **(a)** N2a cells were transfected with either GFP, GFP-Rab21 WT, GFP-Rab21 Q76L, GFP-Rab21 T31N, or GFP-Rab21 T31N Q51G; lysed and subjected to IP with GFP magnetic agarose beads. Whole cell lysates (WCL) and immunoprecipitates (IP) were subjected to Western blot analysis with the indicated antibodies. **(b)** On the left: structure of Rabex-5 (light brown) in complex with human nucleotide-free Rab21 WT (green) (Delprato and Lambright, 2007). On the right: AlphaFold 3 prediction for the interaction between Rabex-5 (light brown) and human nucleotide-free Rab21 Q53G (green) around the indicated residues. **(c)** AlphaFold 3 prediction for the interaction between Rabex-5 (light brown) and either nucleotide-free Rab5 WT or Rab5 G54Q mutant (green) around the indicated residues.

SUPPLEMENTARY FIGURE S7 GFP-Rab5 S34N is partially present at the Golgi complex. **(a)** Representative images of N2a cells transfected with GFP-Rab5 S34N and immunostained with antibodies against GM130 and GFP. Insets on the right show magnification of the boxed areas. Scale bar: 5 µm; insets: 3 µm. **(b)** Quantification of the percentage of cells that present GFP-Rab5 S34N at the Golgi versus the cells that present a cytoplasmic distribution of this protein. The graph shows the mean and standard deviation of three independent experiments (n = 71).
