# Supplementary figures and images for: Rab21 recruits EEA1 and competes with Rab5 for Rabex-5 activation
Source: Front Cell Dev Biol. 2025 May 30;13:1588308. doi: 10.3389/fcell.2025.1588308 (PMC12162657; doi:10.3389/fcell.2025.1588308)

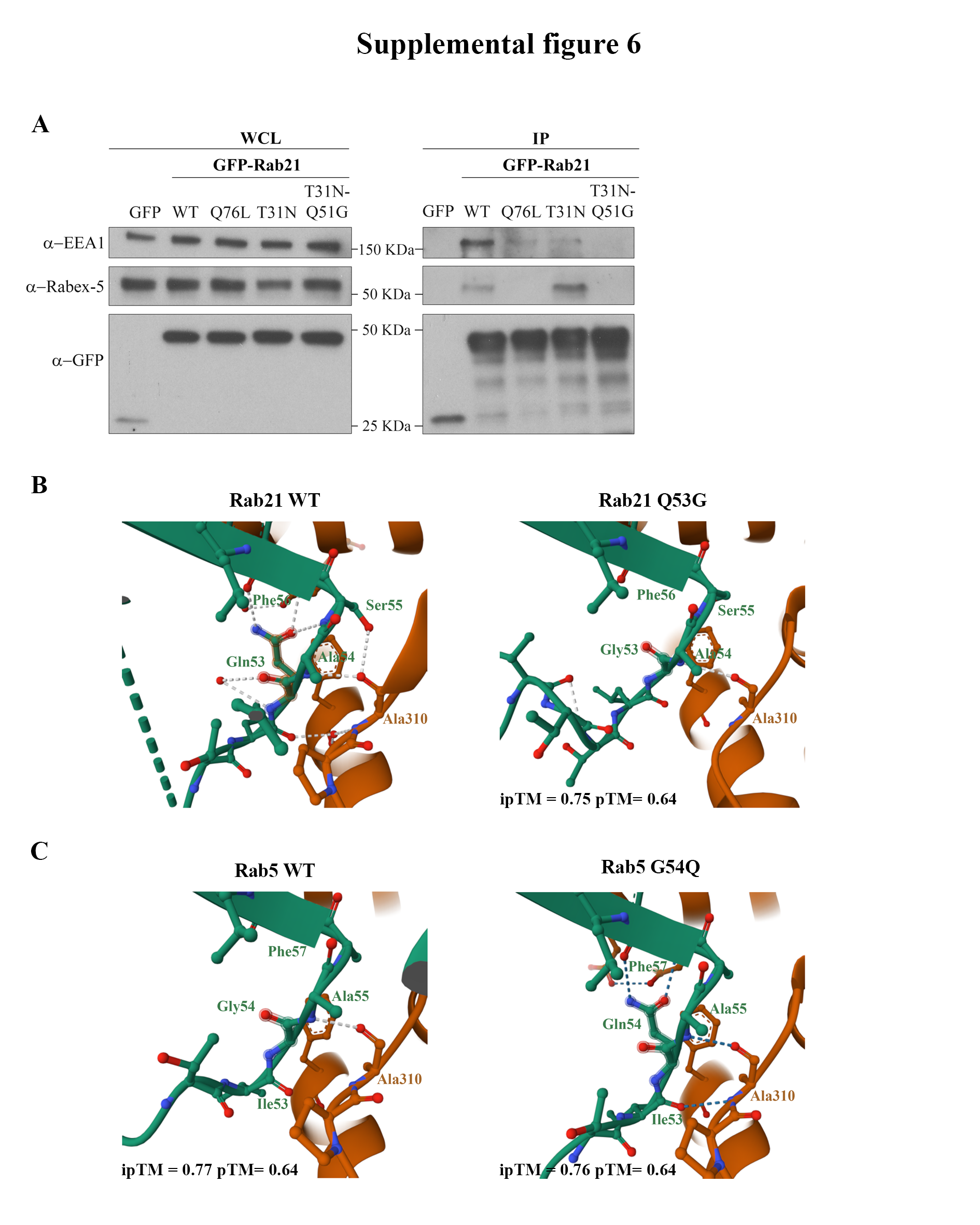

Supplement: Supplementary file 1 [file Image6.tif]

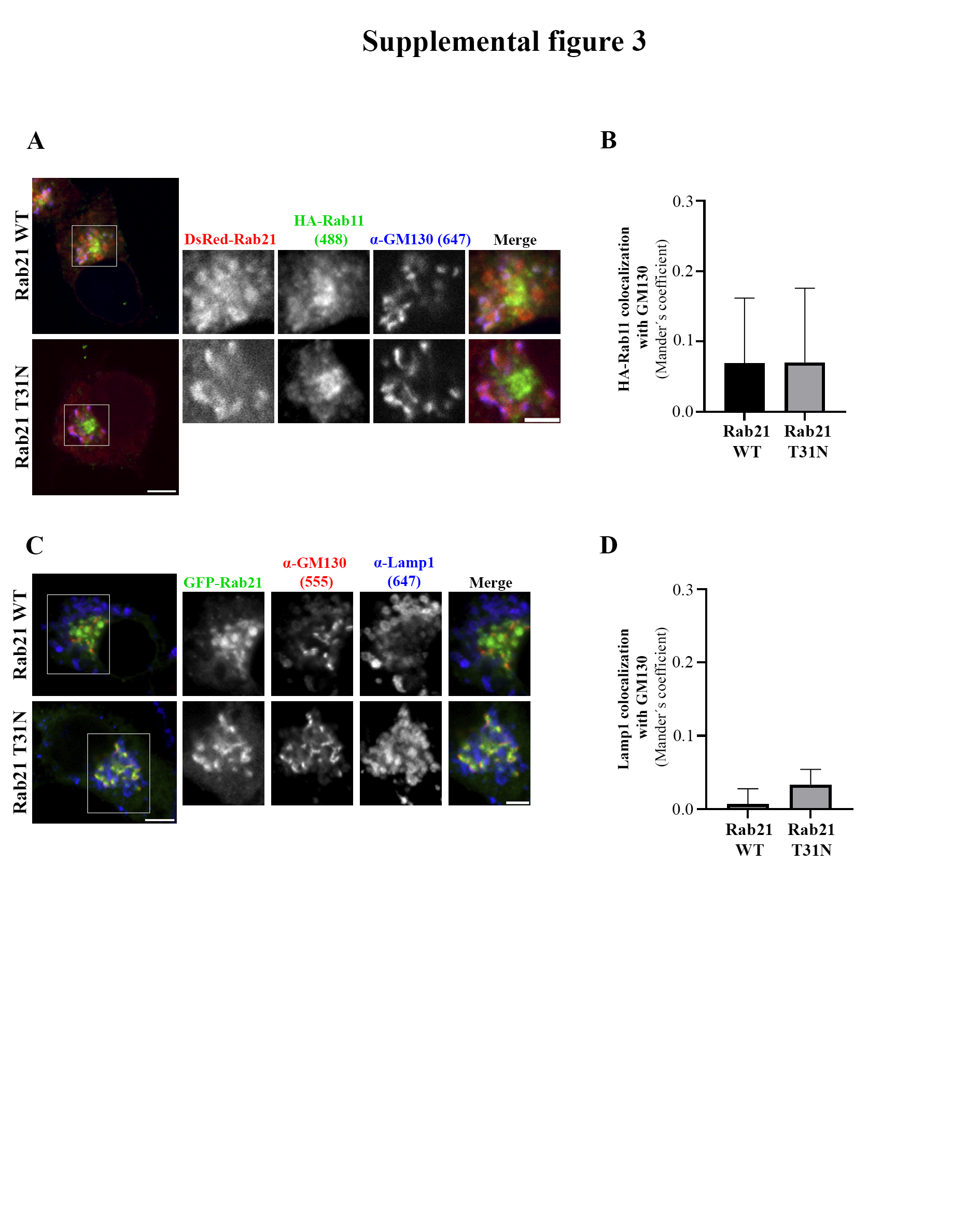

Supplement: Supplementary file 2 [file Image3.tif]

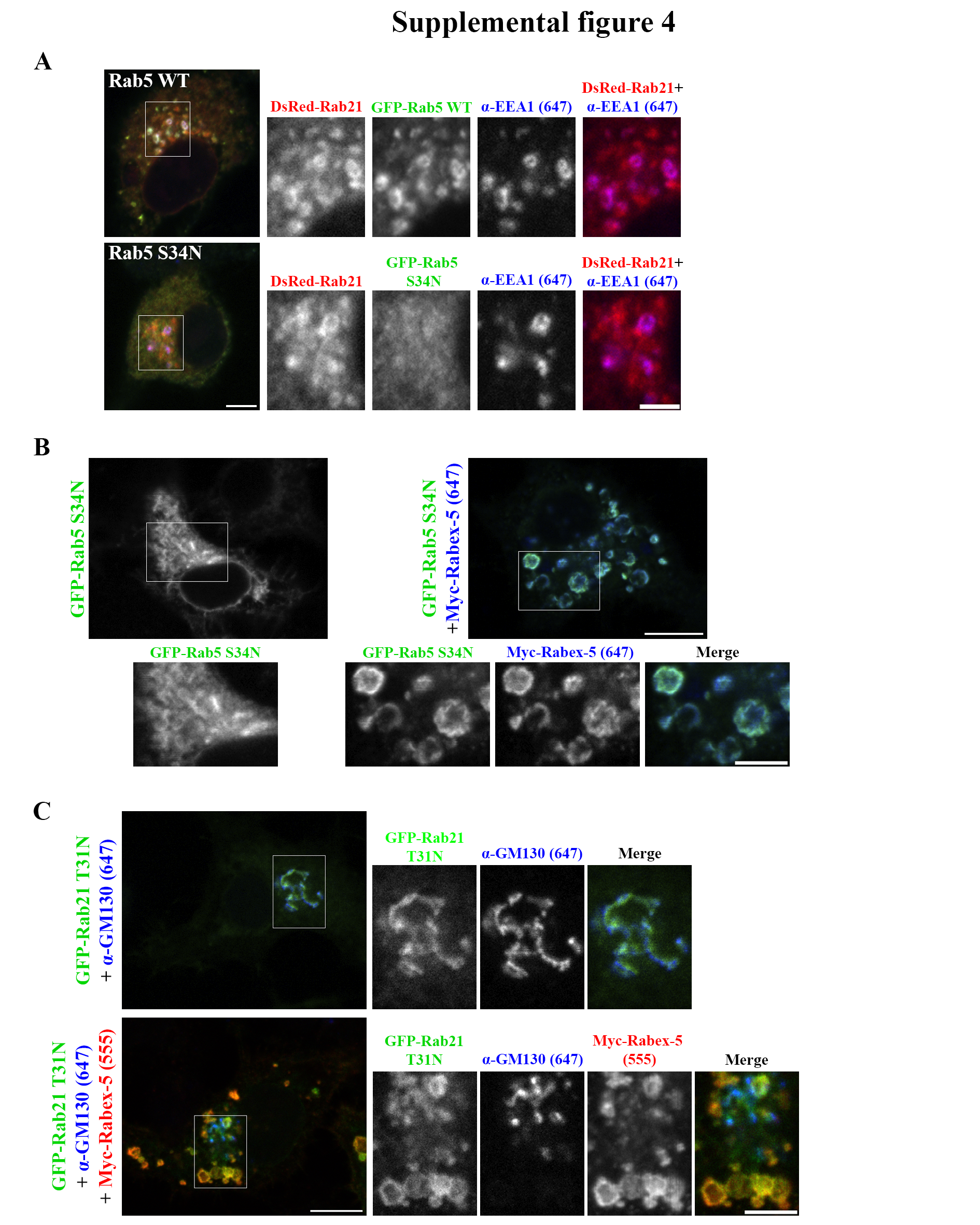

Supplement: Supplementary file 3 [file Image4.tif]

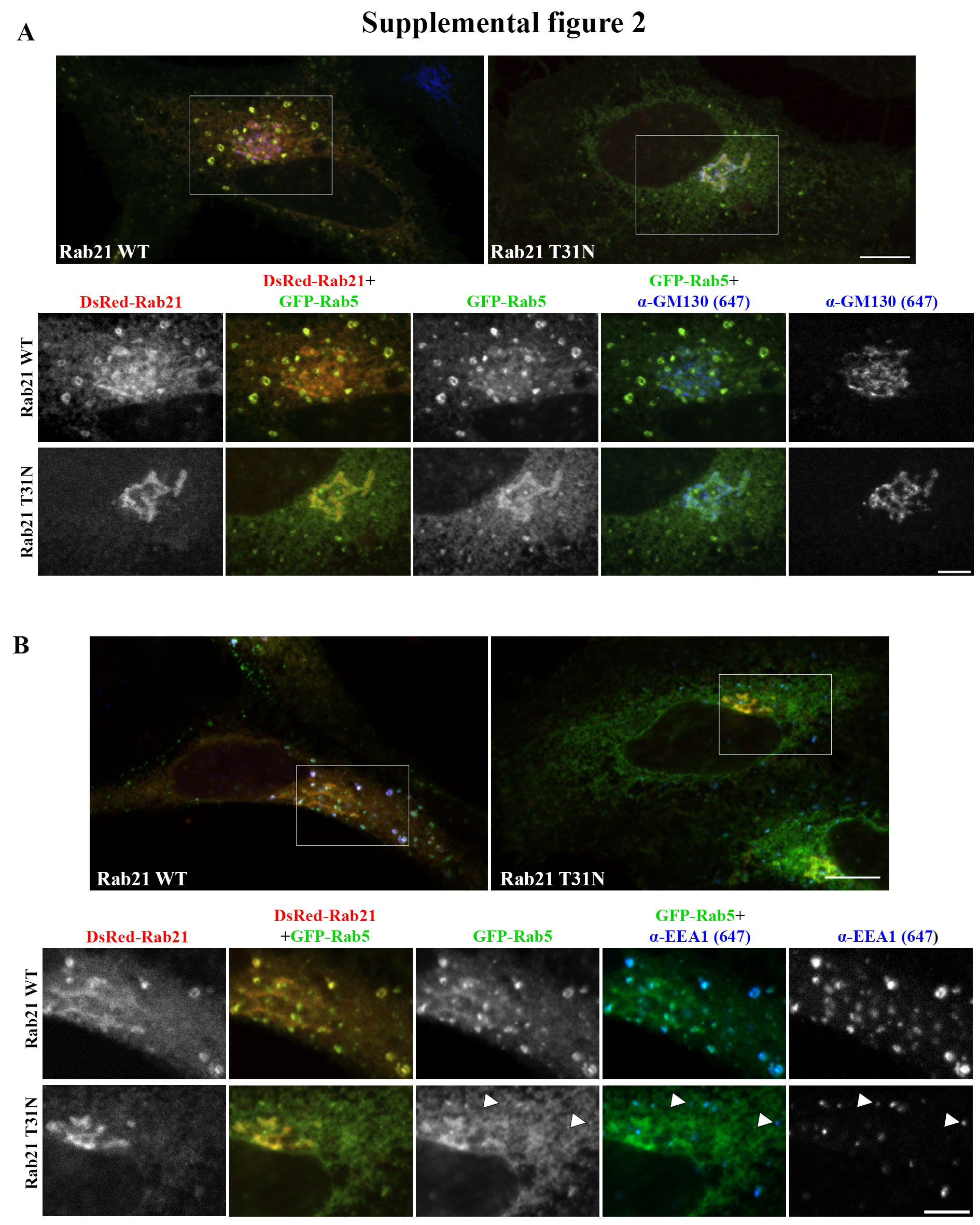

Supplement: Supplementary file 4 [file Image2.tif]

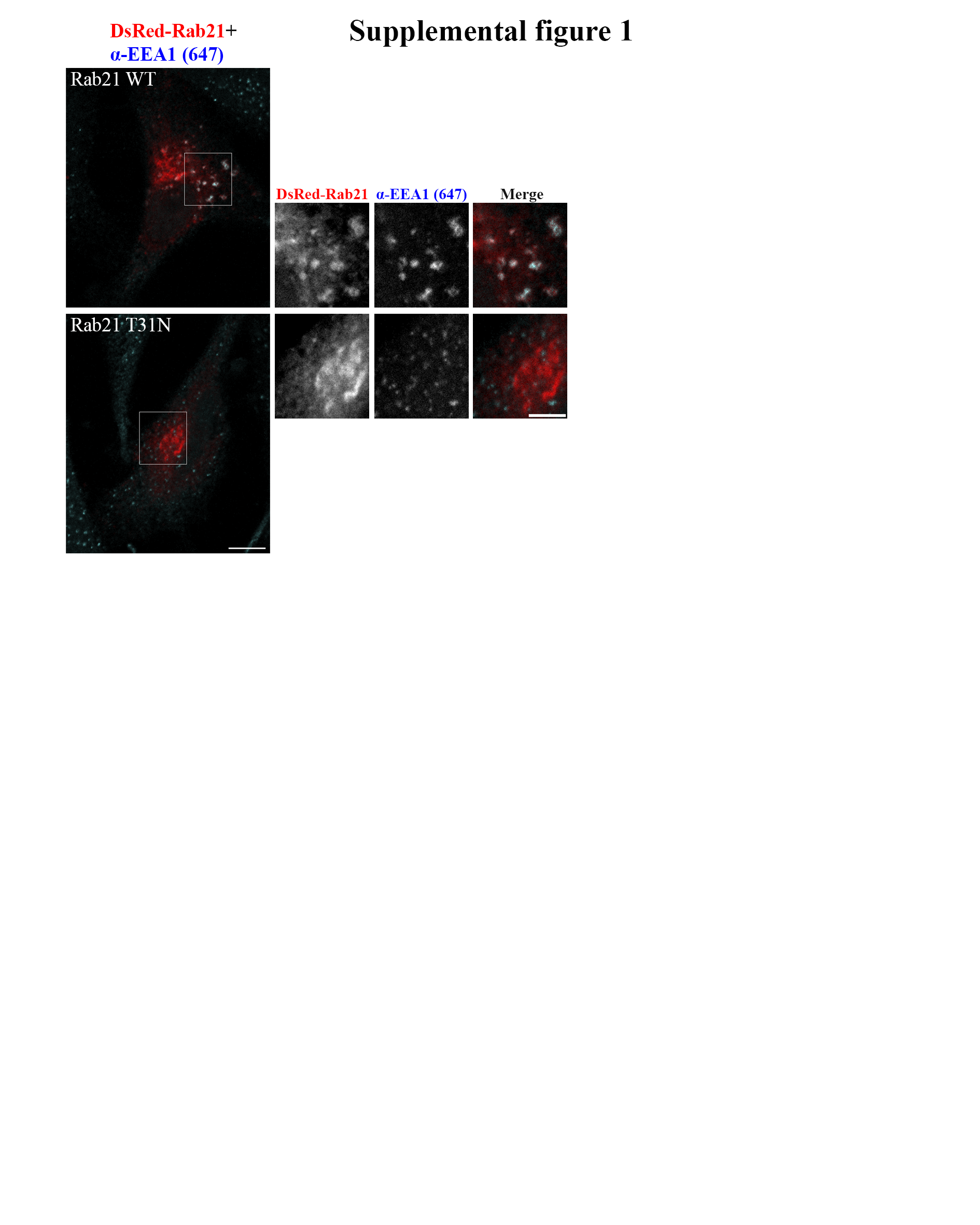

Supplement: Supplementary file 5 [file Image1.tif]

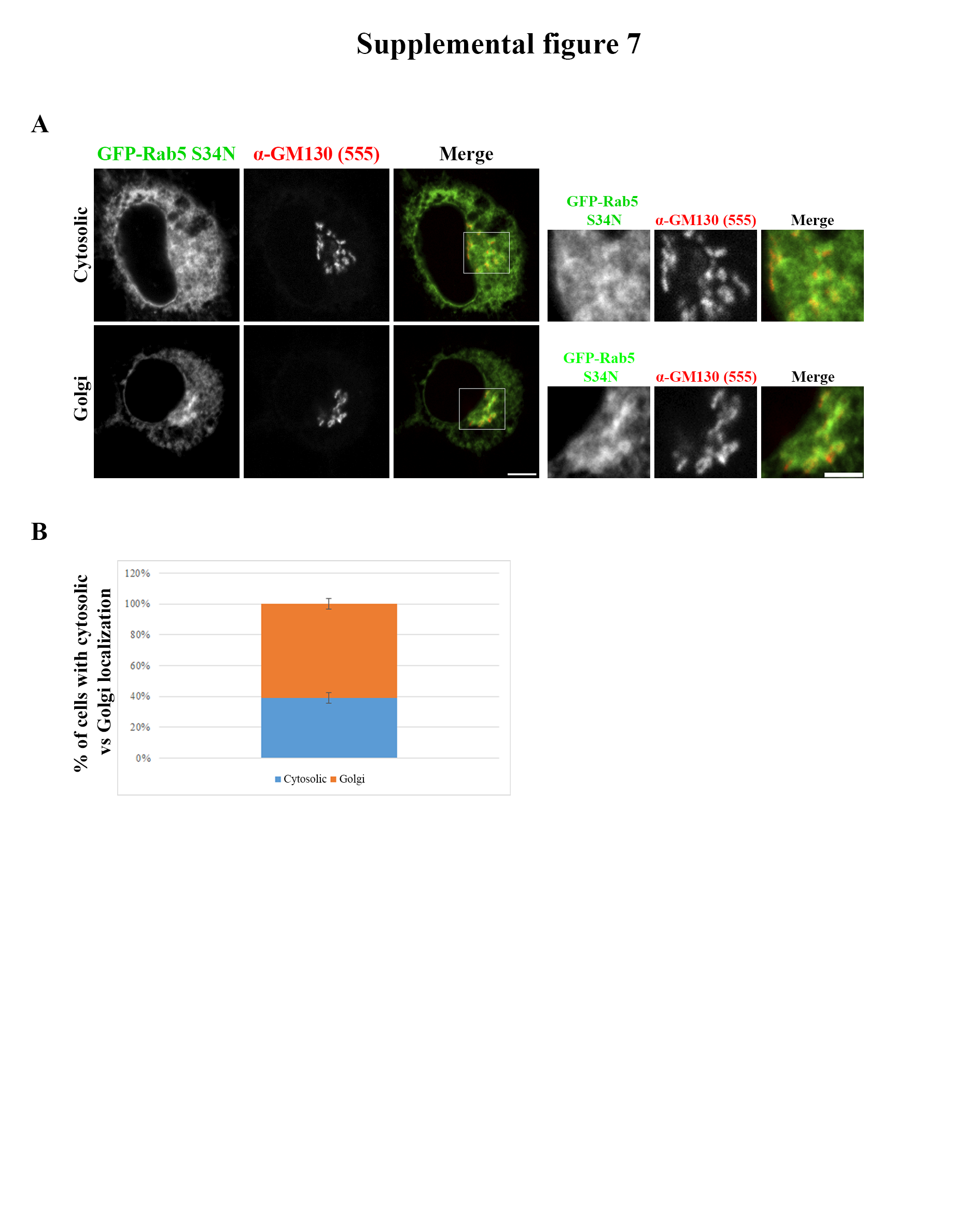

Supplement: Supplementary file 6 [file Image7.tif]

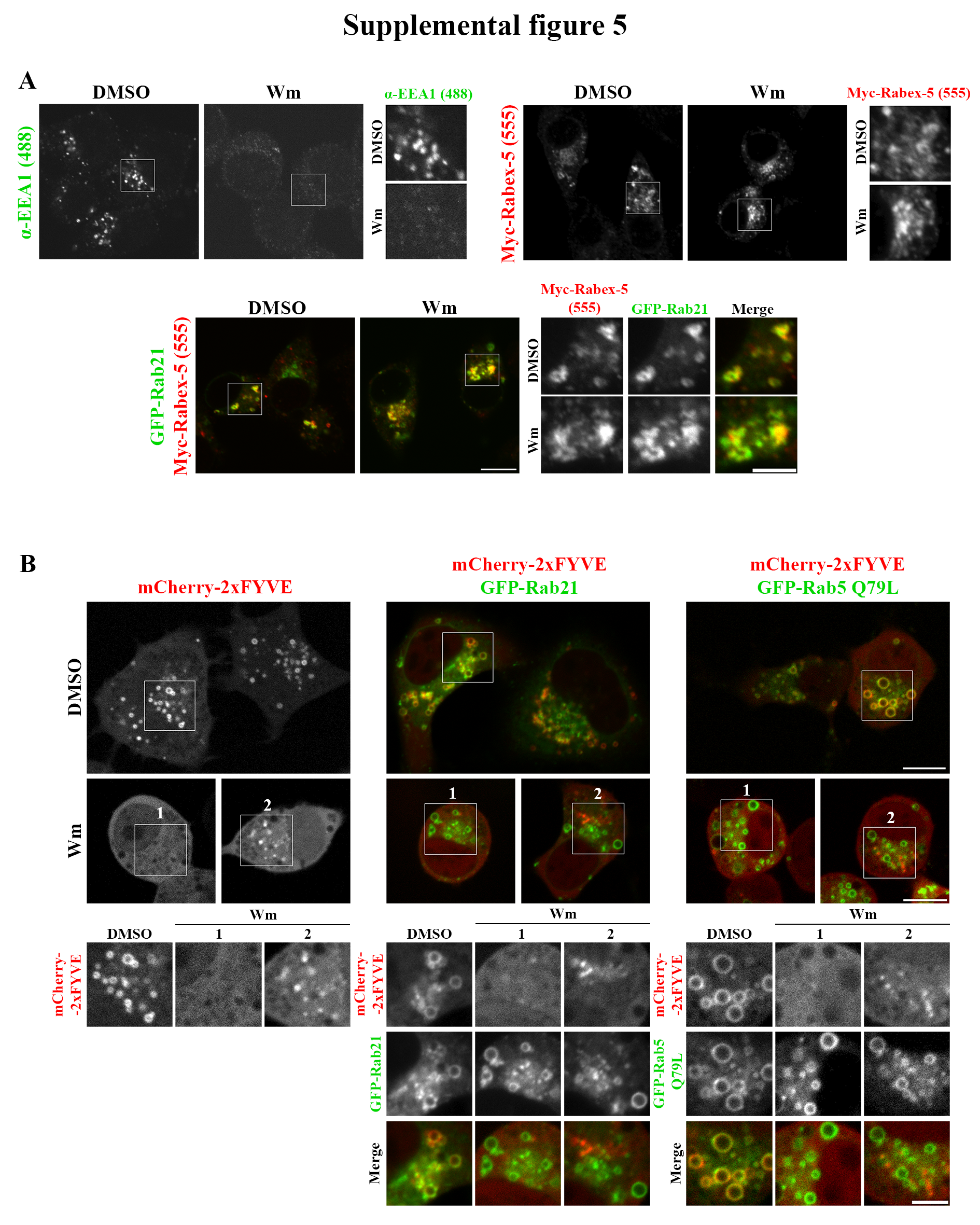

Supplement: Supplementary file 7 [file Image5.tif]
